# The New Challenge for Heart Endocarditis: From Conventional Prosthesis to New Devices and Platforms for the Treatment of Structural Heart Disease

**DOI:** 10.1155/2021/7302165

**Published:** 2021-06-14

**Authors:** Francesco Nappi, Adelaide Iervolino, Sanjeet Singh Avtaar Singh

**Affiliations:** ^1^Department of Cardiac Surgery, Centre Cardiologique du Nord, 93200 Saint-Denis, France; ^2^Department of Cardiovascular Sciences, Fondazione Policlinico Universitario A. Gemelli IRCSS, Rome, Italy; ^3^Department of Cardiothoracic Surgery, Golden Jubilee National Hospital, Glasgow, UK

## Abstract

Infective endocarditis is a sinister condition with considerable morbidity and mortality. Its relevance in the current era is compounded by the increased use of implanted devices such as replacement valves or cardiac implantable electronic devices. These infections are caused by multiple different bacteria with different virulence, pathogenicity, and antimicrobial resistance. Unlike in native endocarditis, the presence of foreign tissue permits sustenance by inflammatory and thrombotic processes as the artificial surfaces promote inflammatory responses and hypercoagulability. Prevention of these infections has been suggested with the use of homografts in combination with antibiotics. Others have attempted to use “low fouling coats” with little clinical success thus far. The use of antibiotic prophylaxis plays a pivotal part in reducing the incidence of prosthesis-related endocarditis. This remains especially crucial with the increasing use of transcatheter heart valve therapies. The widespread use of cardiac implantable electronic devices such as permanent pacemakers, implantable cardioverter defibrillators, and cardiac resynchronization therapy devices has also heralded a noticeable increase in cases of infectious endocarditis affecting complex equipment which can be difficult to treat. Multimodality strategies are needed with input from surgeons and cardiologists to ensure treatment is both prompt and successful, tailored to the individual needs of the patients.

## 1. Introduction

The challenges posed by infections developing in native heart tissue or implanted devices are greater than ever. The current era is characterized by remarkable advances in the field of cardiology with noticeable changes in patient demographics and disease manifestations [1, 2]. This change affects the average patient who is older and frail with increasing comorbidities. During this period as well, strains of *staphylococcus* have supplanted oral *streptococcus* as the most frequent causative organism [3, 4].

The emergence of new cardiac implantable electronic devices (CIEDs) has also heralded a noticeable increase in cases of infectious endocarditis (IE) affecting complex equipment [5]. Likewise, the use of new materials that constitute the armamentarium for transcatheter valve implant (TAVI) performed by percutaneous access has revolutionized these new endovascular platforms for the treatment of structural heart disease. The caveat, however, is that TAVI may be associated with higher rates of IE compared to conventional surgery [6–9].

Another aspect to consider is healthcare-associated infection, which is the result of complicated care in patients who have received the next generation of cardiac devices. Despite the burden of healthcare-associated infections in high-income countries being significantly lower than those occurring in low-income countries [10], there is irrefutable evidence highlighting the need to improve surveillance and infection control practices. This inconvenience in routine patient management has a major impact leading to increased costs, which in the U.S. have been estimated to average around $ 120,000 per patient [11].

Given the constant evolution of infections associated with implants and implantable devices in cardiopathic patients, an evaluation of therapeutic and innovative biomaterials against medical device-associated infections, 2 crucial challenges should be considered: preventive therapy against the most aggressive strains of pathogens and the infection of heart devices including conventional mechanical or biological prosthesis, transcatheter heart valve implantation (THVI), and CIED.

## 2. Microbiology

Infective endocarditis can occur in patients who require the implantation of a cardiac device during initial hospitalization or after discharge from the hospital. *Staphylococcus aureus* represents the most common pathogen among healthcare-associated organisms accounting for around 30% of cases [3, 4]. It is important to underline that in the serial blood culture of patients who develop bacteremia, *Staphylococcus aureus* is found in 70% of the examined.

Concern related to the clinical picture of patients with endocarditis sustained by *Staphylococcus aureus* is the high aggressiveness of the infection which frequently causes complications such as an increased risk of embolism and stroke. Certainly, persistent bacteremia of the pathogen in the blood with distant colonization supports critical illness, systemic sepsis, and death [12]. Other germs implicated in infectious endocarditis developing in high-income countries are oral streptococci which account for about 20% of cases. *Enterococci*, on the other hand, are the cause of an additional 10% of bacterial colonization [13].

With the increase in infectious endocarditis supported by *Staphylococcus aureus*, infections caused by *coagulase-negative staphylococci* (CoNS), as for example *Staphylococcus epidermidis, Staphylococcus lugdunensis*, and *Staphylococcus capitis*, have assumed a particular menace. One of the characteristics that make *staphylococci CoNS* potentially very harmful is that they are ubiquitous and skin commensal, so they can easily colonize infusion catheters and indwelling devices [14–16].

The concern related to early endocarditis caused by *staphylococci CoNS* also concerns the infectious processes that develop in the materials that make up the valve bioprostheses and CIEDs. In implanted biomaterials, bacterial colonization of pathogens such *Staphylococci CoNS* can reach up to 10%, thus, characterizing device infections (bioprosthesis and CIED) for two reasons [15, 16]. The first relates to the early onset of these infections in the first year following the initial procedure [17]. The second is the frequent resistance to methicillin after bacterial colonization with foci of infection sustained by the *Lugdunensis* strain of *Staphylococcus*, endowed with a particular aggressiveness towards biological tissues and biomaterials [18].

There are other opportunistic pathogens, zoonotic bacteria, and fungi that can lead to the development of particularly insidious infections. Bacteria included in the HACEK group (*haemophilus*, *aggregatibacter*, *cardiobacterium*, *Eikenella corrodens*, and *kingella)* are responsible for a low percentage of infections (3% of cases). The peculiarity that makes these pathogens insidious is the slow growth and the frequent colonization in the oropharynx, and they can adhere to the cardiac devices of immunosuppressed patients [19].

Unusual pathogens causing IEs include Gram-negative bacteria (e.g., *Acinetobacter* spp and *Pseudomonas aeruginosa*), *Legionella* spp, *Mycoplasma* spp, and *Tropheryma* whippelii [19]. Particular attention should be given to infections supported by fungi, generally, *Candida* or *Aspergillus*, which although infrequent, are however lethal and aggressive. They occur mainly in immunosuppressed patients or after cardiac surgery procedures, especially in patients who have received prosthetic valves or devices for the treatment of arrhythmias [20].

## 3. Mechanisms Sustaining Infection in Cardiac Device

The infection that occurs on cardiac devices is sustained by reactive inflammatory and thrombotic phenomena. Unlike living tissues, which are actively resistant to the inflammatory and thrombotic processes, artificial surfaces promote inflammatory and hypercoagulability reactions through a complex series of interrelated pathological processes including leukocyte adhesion, complement activation, and protein adsorption. The phenomenon is also supported by the fixing of red blood cells and platelets with the formation of thrombin [ 21, 22]. Thus, infection of the biomaterial develops along 3 stages.

In the first stage of bacteremia, pathogens commonly enter the bloodstream using three main routes of access: the mouth, the gastrointestinal and urinary tracts, or the skin. The latter includes venous catheters or the use of invasive medical devices or surgical procedures.

The second phase includes adhesion. Among the priorities of the endothelial lining of the heart structure in physiological conditions include resistance to bacterial adhesion. This protection is absent in the damaged endothelium and in the artificial surfaces which are therefore exposed to bacterial adhesion, through superficial adhesins. These are specific proteins whose main action lies in attachment to the host's extracellular matrix proteins. The process is promoted by fibrin and platelet microthrombi [23]. The phenomenon of colonization of biomaterials by pathogens is mediated by the adhesion of microorganisms which give rise to cycles of bacterial proliferation associated with the establishment of a thrombosis process. This has been substantiated by several reports that the [24–27] fibronectin-binding protein and staphylococcal clumping factors A and B play a crucial role in causing bacterial adherence and pathogenicity [24]. The adhesion can lead to the colonization of microorganisms and the formation of infected vegetations on the surface of biomaterials [25, 26]. Finally, the production of polysaccharide biofilm sustains both bacterial persistence and tolerance to antibiotic treatment [27].

The third phase is the recruitment of monocytes and activation of the inflammatory cascade leading to the formation of mature vegetation on the artificial surface [28]. This phase is coupled with the thrombotic process. During exposure of human blood plasma to polyethylene, plasma proteins play a crucial role in thrombus formation as they are subject to rapid surface adsorption, thus, having a role in modulating subsequent reactions [29]. In detail, fibrinogen is the prime actor and is one of the first plasma proteins to deposit on material surfaces. Subsequently, other adhesive proteins, including fibronectin and von Willebrand factor, also migrate to the surface of the biomaterials and, together with fibrinogen, mediate platelet adhesion. Once adhered to the surface, platelets are activated and release adenosine diphosphate, thromboxane A2, and other agonists leading to cascade recruitment of additional platelets onto the surface of the materials. In this phase, the process is irreversible because, to the adsorbed fibrinogen, other proteins are associated, constituting the components of the contact system, including factor (f) XII, high molecular weight kininogen, prekallikrein, and fXI [29].

Many of the pathogens that colonize the infectious fields of biomaterials used for the treatment of heart disease consists of common bacteria such as *staphylococci*, *streptococci*, and *enterococci* but may also include infrequent pathogens such as *Candida* and *Pseudomonas*; all of which produce biofilms. Biofilms work in three directions: by promoting the integration of pathogen populations within the extracellular polysaccharide matrix by forming a dense slime, by activating cell-to-cell chemical transmission, and by promoting synchronized gene expression that leads to the assembly and maturation of microorganisms. Elgharably et al. [30] reported that once the biofilm is rooted in the extracellular matrix, it protects the bacteria from the host's immune defenses, preventing both antimicrobial efficacy and concealment of persistent and resistant bacteria. Chung and Toh [31] have recently shown that the role of biofilm-inducing pathogens is crucial in increasing virulence in infections that develop in cardiac devices caused by staphylococcus strains.

## 4. Cardiac Device and Infection

Infective endocarditis affects biomaterials of cardiac devices such as valve bioprostheses, percutaneous transcatheter heart valves, and cardiac implantable electronic devices.

**Figure 1:**
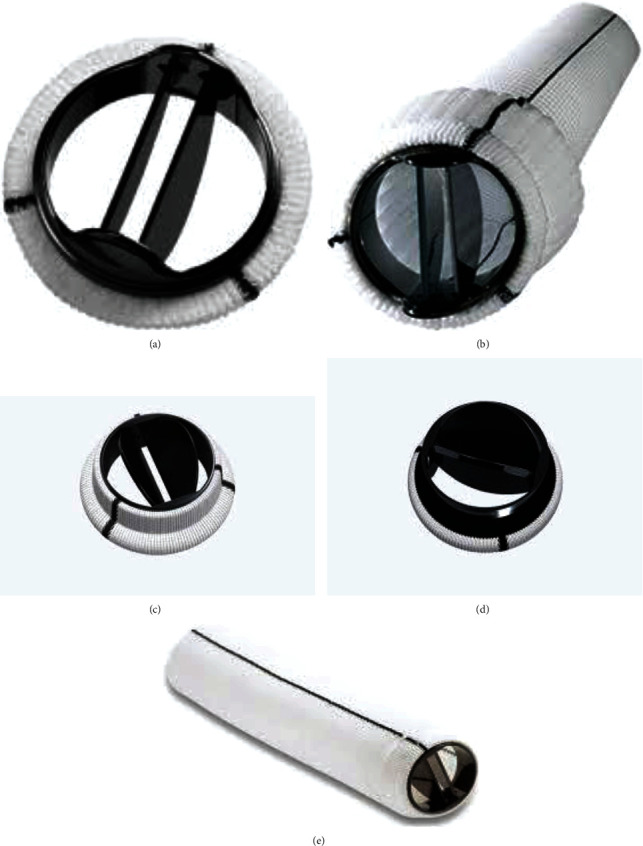
Conventional mechanical prosthetics. The device can be single or assembled with a Dacron prosthesis (valved Dacron tube). (a) St Jude Medical Regent bileaflet mechanical valve. (b) Valsalva mechanical valved graft (St. Jude Medical, Minneapolis, MN). (c) Carbomedics Aortic Mechanical Valve. (d) Bicarbon Aortic Valve (LivaNova, PLC, London, UK). (e) Carbomedics mechanical valved graft (LivaNova, PLC, London, UK).

**Figure 2:**
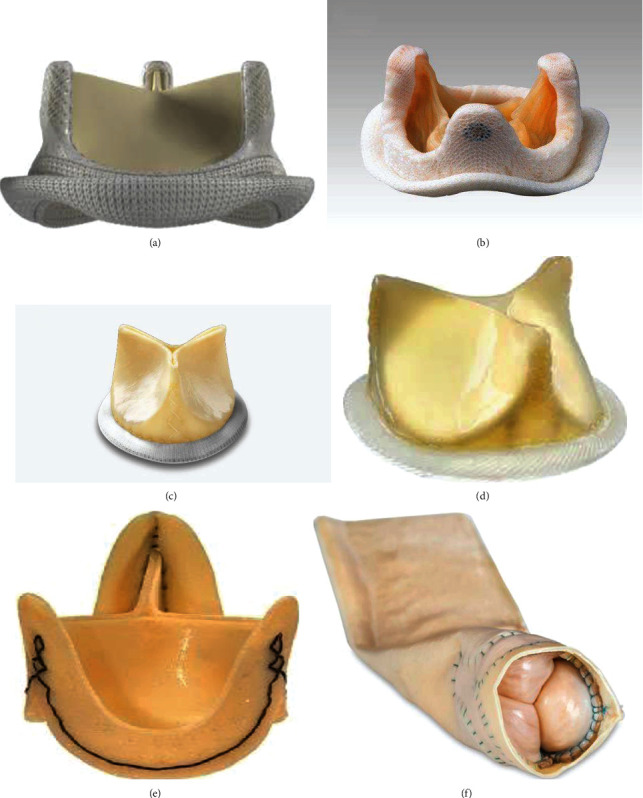
Last generation of stented/stentless xenograft prosthesis constituted of pericardial or porcine leaflet. The device can be single or assembled as a bioconduict. The biological prosthesis includes as biomaterials valve leaflets which can be of calf or porcine pericardium and a polytetrafluoroethylene stent. The leaflets are inserted on the circumference of polytetrafluoroethylene annulus. The 3 leaflets are separated by 3 commissures. (a) Carpentier-Edwards Perimount stented aortic (Edwards Lifesciences Inc., Irvine, California, USA). (b) Medtronic Mosaic stented aortic (Medtronic, Minneapolis, Minnesota). (c) Mitroflow aortic bioprosthesis (models 12A/LX; LivaNova, PLC, London, UK). (d) St Jude's Trifecta aortic bioprosthesis (St. Jude Medical, Minneapolis, MN). (e) Freedom Solo Stentless aortic valve (LivaNova PLC, London, UK). (f) Bioconduit (BioIntegral Surgical, Inc.).

Valve prostheses include both mechanical and biological prostheses. Mechanical prostheses are mainly composed of metal and pyrolytic carbon. On the other hand, biological prostheses include xenografts that can be formed from different types of bovine or porcine pericardial biomaterial and homografts obtained from the human corpse during organ harvesting. The next generation of conventional stented/nonstented xenograft and mechanical prostheses is reported in Figures 1 and 2.

Heart valve surgery can be fulfilled with the use of either a mechanical or a biological prosthesis. However, there are several disadvantages to the use of these biomaterials. The benefit related to the use of mechanical valve prostheses, which are produced with pyrolytic carbon, is the greater durability; however, the disadvantage linked to their implant is the prolonged lifelong anticoagulation treatment and the risk of thromboembolism. The pyrolytic carbon prostheses are preferentially implanted in younger subjects, with a life expectancy of more than 15 years [32]. Conversely, in bioprosthetic devices, the biggest drawback is the risk of prosthetic-valve deterioration and failure, which is caused by the processing of the biomaterials that make up these devices [33, 34].

Concern related to the use of biomaterials, which is the main limitation of biological prosthesis, is the achievable rejection due to the immune response of the recipient. These are, therefore, decellularized to eliminate all donor cells from the matrix. However, the decellularization process can significantly modify the functional characteristics of the extracellular matrix, drastically altering its biochemical and biomechanical properties. For this reason, the average duration of biological valves, which requires a short anticoagulant time of three months [32], is approximately 15 years. Degenerative processes can arise due to the chemical cross-links that are necessary to confer stability and durability to the collagen fiber after the decellularization process [35, 36]. Crosslinking is often achieved using glutaraldehyde which is an inexpensive water-soluble reagent but capable of ensuring stable crosslinking. However, the slow release of unreacted glutaraldehyde, present in the matrix after treatment, can cause different inconvenient such as cytotoxicity, inflammatory response, and calcification [37, 38].

Infective endocarditis has a prevalence of 51% for the involvement of the aortic valve. Among these, in 59% of cases, the infection affected the implanted prosthetic valve biomaterial, and in 68% of cases, it was characterized by an accentuated invasiveness. The mitral valve is involved in 30.7%; while in 29%, the infectious processes were limited to the prosthesis biomaterial although in 35% of cases, and it had spread to nearby cardiac structures [21, 22, 39–41].

**Figure 3:**
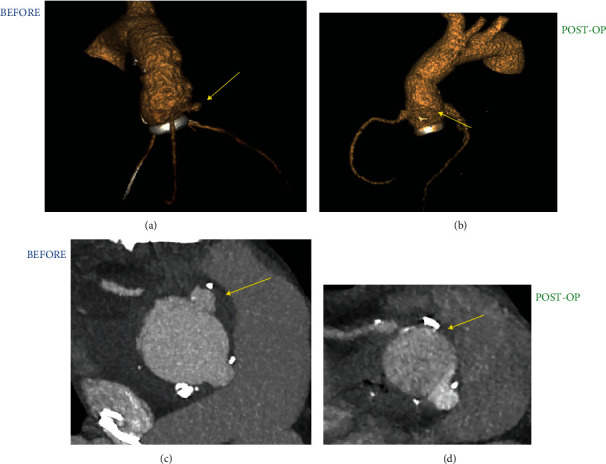
Infectious process involving a valved tube consisting of mechanical prosthesis assembled with a Dacron vascular graft. (a and c) Recurrent false aneurysm (arrow yellow) after implantation of Carbomedics mechanical valved graft (LivaNova, PLC, London, UK). (b and d) The infection of cardiac device is successfully treated with the use of valsalva mechanical valved graft (arrow yellow) (St. Jude Medical, Minneapolis, MN).

Conventional mechanical or stent/stentless xenograft prostheses are the most commonly used for the surgical treatment of infectious heart disease; however, the use of allogeneic and autologous tissues is considered in selected cases. The resistance by cardiac surgeons to the use of allogeneic and autologous tissue, as well as for the choice between the prosthesis in biomaterial or pyrolytic carbon as the ideal substitute for use at the site of infection, probably derives from the lack of randomized clinical trials (RCTs) to support the observational studies [39–44]. The Stanford group [45] studied the impact of selecting prosthetic valves for the treatment of left-sided endocarditis. Between 1964 and 1995, 306 patients underwent left-sided valve replacement, 68% of whom had a native heart valve infection while in 32% of cases the infection involved the biomaterials of the implanted prostheses. The authors revealed that the risk of reoperation for infection relapse was similar among mechanical (2.1%) versus bioprosthetic valves (2.3%) at 5 years and a slightly increased risk for mechanical prosthesis (0.5%) compared to stented xenograft (1.1%) beyond 5 years [45] (Figure 3).

**Figure 4:**
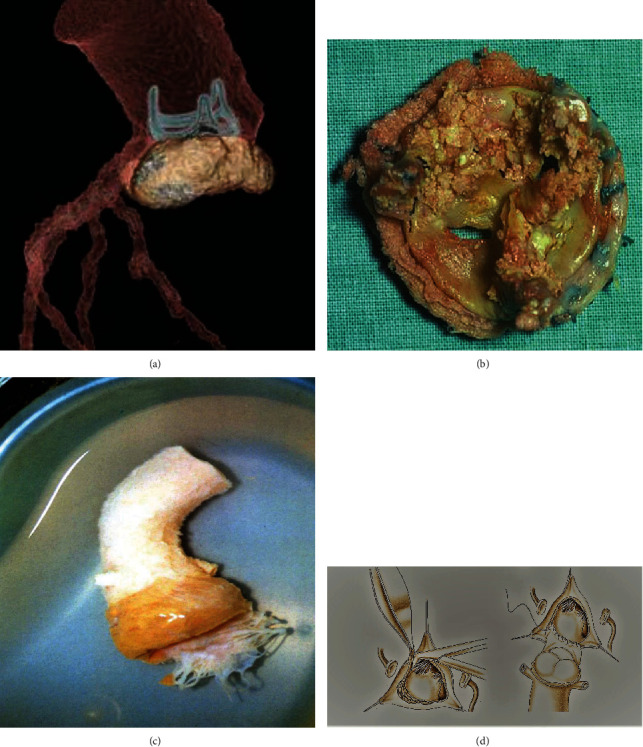
Infectious process with large periprosthetic abscess involving biological prosthesis. (a) Abscess formation of conventional stented bioprosthesis. (b) The prosthesis is removed with vegetations. (c) Biological living tissue explanted and cryopreserved (homograft) is used for aortic root reconstruction and for repair of mitro-aortic curtain. (d) The infected prosthesis is removed with aggressive debridement of all infected and necrotic tissue. The coronary ostia are prepared for the reconstruction of aortic root.

The 2015 report from the Society of Thoracic Surgeons [ 15] recorded an increasing use of conventional biological prosthesis compared to mechanical ones. The biomaterial was used in 73% of patients in native heart infection and in 27% of reoperations to replace infected mechanical or biological implants. On the other hand, in primary infection, a cryopreserved homograft was used in only 2.5% of patients compared to 68.7% of these in which a biological valve was used. Therefore, a reversal in the trend regarding the use of homograft is evident both in native cardiac infections and in those occurring on previously implanted mechanical or biological prosthetic surfaces. Allogenic tissue is most commonly used in the presence of reinfection of biomaterials or artificial surfaces such as in the case of mechanical prostheses compared to primary infection (32.2% vs. 7.0%, *p* < 0.0001) [46–53] (Figure 4).

### 4.1. Prevention

Since the biomaterials commonly used in cardiological devices do not have systems to counteract the harmful effects of the biofilms produced by bacteria, prevention is entrusted to the use of antibiotic therapy.

Several studies strongly supported the use of homografts in extensive cardiac and vascular structural infections, both in native and prosthetic valvular disease [ 54–57]. Evidence suggests that the use of allogeneic tissue has antibacterial activity despite long-term storage for 5 years. For example, combinations of antibiotics using gentamicin, piperacillin, vancomycin, metronidazole, amphotericin B, flucloxacillin, meropenem, tobramycin, and colistin, when applied during allogeneic tissue processing, lead to significant resistance to infection. In fact, allogeneic tissues when implanted to replace ascending aortas have developed a better resistance to bacterial infection sustained by staphylococcal bacteria such as *Staphylococcus epidermidis* and *Staphylococcus aureus*. It should be noted that bacterial contamination of the aortic homograft was less in the tissue of the vascular conduit than in the aortic valves [54]. Furthermore, allogeneic tissues treated with flucloxacillin have proven to be effective in resisting infections caused by *Pseudomonas aeruginosa*. Instead, the use of meropenem and colistin improved resistance to infections caused by *Escherichia coli*^54,55n^.

Another study demonstrated the efficacy of antibiotic pretreatment of the implanted cryopreserved allogeneic tissue that led to a significant decline in infection relapse [56]. The same result has not been recorded with the use of conventional prostheses or as results of grafting with Dacron prosthesis, although the risk of developing a vascular grafting infection is reduced after pretreatment of Dacron with antibiotics [56].

The action of the antibiotic/fibrin combination leads to a favorable effect mediated by the progressive release of antibiotics so as to prevent early recurrent infection [57]. Based on these findings, more effective concentrations of *β*-lactam antibiotics have been used both to increase anti-infectious activity and to provide additional immunity in case of recurrence of the infection [57].

The favorable support of antibiotics in the prevention of autologous and allogeneic tissue infection has been widely demonstrated over the past thirty years. Important studies have reported few cases of infection, with a percentage between 2% and 5%, after the use of homotransplantation and autograft they have been successfully treated from a medical point of view [ 48–50, 58–60].

There is no doubt that in today's practice, the focus should be on identifying new approaches to prevent bacteremia and strategies aimed at counteracting the adhesion of microorganisms to the surface of materials [61]. A new technological track to pursue is that of the so-called low fouling coats. These innovative technologies can be applied to biomaterials can prevent the interaction of bacteria with prosthetic surfaces. They use long-lasting bactericidal coatings which, although promising, have not shown efficacy in clinical practice.

The most obvious example of failure to translate innovation into clinical application is the use of the Silzone valve (St. Jude Medical, St. Paul, Minnesota). This pyrolytic carbon prosthesis consisted of a silver-coated sewing ring and was enthusiastically implanted for three years starting in 1997 because it provided an antibacterial coating. The results of the follow-up mitigated the initial enthusiasm for the high rate of thrombosis and perivalvular leak manifested in the Silzone prosthesis recipients [62, 63]. The major concern is related to the fact that any modification of the regulation for the use of mechanical and biological bioprostheses requires a long process for approval, efficacy, and safety must be guaranteed by a significant margin to avoid the failure of new products.

Several studies have focused on the limitation of antibiotic prophylaxis on the incidence of IE in the presence of devices. Opinions differ between public health agencies in various countries.

In France, restrictions on the use of oral therapy with antibiotics have been in place since the early 2000s where the administration of drugs is limited to high-risk cases. An analysis involving the different French regions did not show substantial changes in the incidence of heart infections although restrictions on taking oral antibiotic prophylaxis were not modified. The data reported from this study confirmed no significant change in the incidence of oral streptococcal infection [64, 65].

**Figure 5:**
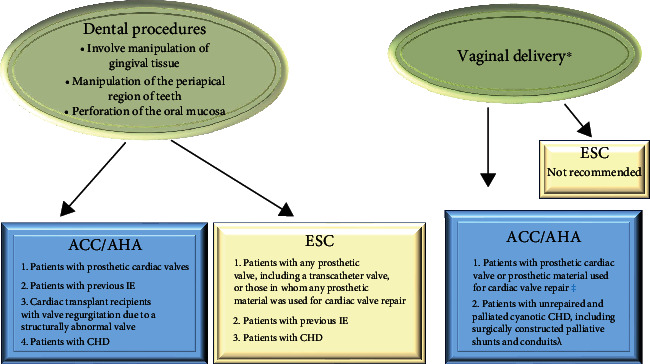
ACC/AHA and ESC Guidelines on Use of Antibiotic Prophylaxis for the Prevention of Infection of Heart Structure.

In 2007, the position of the American College of Cardiology/America Heart Association (ACC/AHA) [66] was to limit antibiotic prophylaxis to the presence of valve prosthesis biomaterials, CHD and previous episodes of IE, as well as in cases whereby heart transplants were required due to structural heart valve disease. Data from a subsequent study processed in the Rochester Epidemiology Project showed no increase in the incidence of IE. On the other hand, Desimone et al. [67, 68] reported a decrease in the incidence of infections related to the aggressive strains of Streptococcus Viridans, recording a rate of 3.6% per 100,000 person-years by analyzing the period 1999-2002 compared to 1.5% per 100,000 person-years for the period 2011-2013 [67, 68]. The same guidance was provided by two other studies that supported a lack of evidence for a change in incidence after the publication of the ACC/AHA recommendations [69, 70] (Figure 5).

The data reported by 2 nationwide epidemiological studies, one from the United States and the other from the United Kingdom, are in clear disagreement with the French experience. They provide food for thought. The study by Pant et al. [71] based on Nationwide Inpatient Sample demonstrated a significant statistically increased incidence of IE linked to streptococcal infection, even if in parallel they were not considered significant increases in total hospitalizations or Staphylococcal infections. In the UK, we can observe two divergent situations. In March of 2008, under the national guidance impulse, the use of antibiotic prophylaxis was not recommended, and immediately after the provision, the initial analysis carried out did not indicate any increase in IE [72]. Data emerged in a 2015 [73] report of an extended analysis that examines hospital discharge diagnoses up to 2013 in the NHS. The use of antibiotic prophylaxis has been drastically reduced to 1/5 of the doses following the introduction of new guidelines issued by the UK's National Institute of Health and Care Excellence Guidelines. There was a significant increase in correspondence in the number of heart infections equal to 0.11 cases per 10 million people per month. Statistical analysis was performed in June 2008, at the moment of the change, despite the lack of microbiological to confirm that the organism responsible for the increase of the infection was the oral Streptococcus [73]. The absence of data from randomized studies resulted in reliance on a retrospective analysis with its inherent biases, among other things, by the increase in the number of implanted devices. In fact, these data are retrospective and cannot establish a causal link between the restriction of antibiotic prophylaxis and the incidence of IE. The need for randomized trials in a large population that has implanted cardiac devices would certainly help to address this current conundrum.

## 5. Infection in New Cardiac Device Platforms

In the 21st century, new platforms for the treatment of structural heart diseases have emerged, and heart infections have therefore continued to evolve, reaching a rate of increase of >25% in cases needing further treatment.

### 5.1. Transcatheter Heart Valve Implantation

The new transcatheter heart valve (THV) prostheses that use a minimally invasive procedure either trans-thoracic or percutaneous approach have radically changed the treatment of structural heart valve disease. The transcatheter heart valve therapy (THVT) in the last 10 years, after use in the first instance for more fragile patients with multiple comorbidities and high surgical risk, has been successfully extended to intermediate and low-risk patients. It is important to note that, due to the characteristics of fragility and comorbidity in which there are many patients, the increased risk of bacteremia and infective endocarditis is not negligible [9].

The devices available on the market are the balloon, self, and mechanical expandable systems. The first two commercial transcatheter heart valve platforms that were approved for use in high or extreme risk surgical patients were the Edwards Lifesciences intra-annular balloon-expandable transcatheter heart valve platform [74–77] and the Medtronic supra-annular self-expanding transcatheter heart valve platform [78–80].

**Figure 6:**
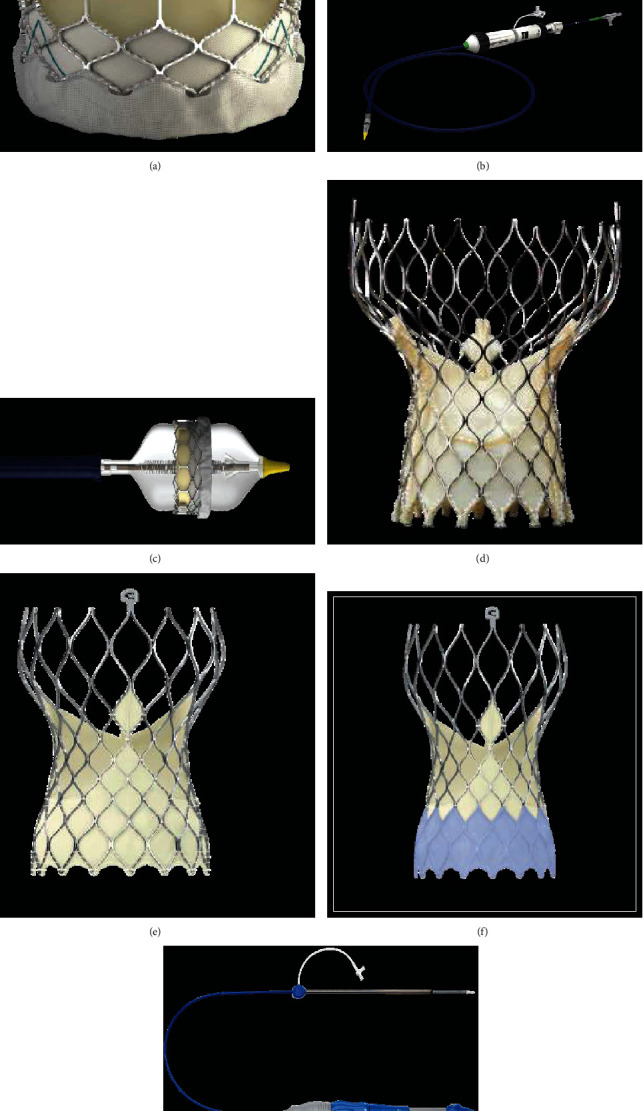
Balloon expandable THV. (a–c) The SAPIEN 3 balloon expandable is constituted by cobalt-chromium alloy frame valve with bovine pericardium leaflets. A polyethylene terephthalate fabric skirt on the distal section of the frame serves for PVL serves prevention. The device is introduced in the market in the following sizes: 20 mm, 23 mm, 26 mm, and 29 mm (a). The Commander Delivery System is 14 F expandable introducer sheath compatible for 20-26 mm valves and 16 F expandable introducer sheath compatible for 29 mm valves (b and c). (d–g) Self expandable THV. The bioprosthesis is manufactured by suturing 3 valve leaflets and a skirt, made from a single layer of porcine pericardium, onto a self-expanding, multilevel, radiopaque frame made of Nitinol (d–g). CoreValve (d), Evolut R (e), Evolut PRO (f) in the following sizes: 20 mm, 23 mm, 26 mm, and 29 mm. ©. The loading System (g). The outer diameter of the catheter is 15 Fr (AccuTrak™ stability layer) and 12 Fr, and the outer diameter of the valve capsule is 18 Fr. The catheter can be used for femoral, subclavian/axillary, or ascending aortic (direct aortic) access sites.

The next generation of balloon-expandable valve is the SAPIEN 3 system (Edwards Lifesciences). The SAPIEN 3 THV (Edwards Lifesciences) is a balloon-expandable, transcatheter aortic heart valve replacement device system that consists of a cobalt-chromium alloy frame and bovine pericardium leaflets [77] (Figures 6(a)–6(c)). The alternative to balloon-expandable transcatheter bioprosthesis is the CoreValve System comprising of a self-expanding nitinol frame and tri-leaflet porcine pericardial valve (CoreValve, Medtronic) [80] (Figures 6(d)–6(g)).

Recently, 2 other self-expandable devices have been evaluated in randomized controlled trials: the Portico (Abbott Structural Heart, St Paul, MN, USA) Self-expanding intra-annular Re-sheathable Transcatheter Aortic Valve System [81] and the ACURATE neo (Boston Scientific, Marlborough, MA, USA) [82] transcatheter heart valve which showed efficacy and safety when compared to the Sapien 3 balloon-expandable THV or other self-expandable devices.

**Figure 7:**
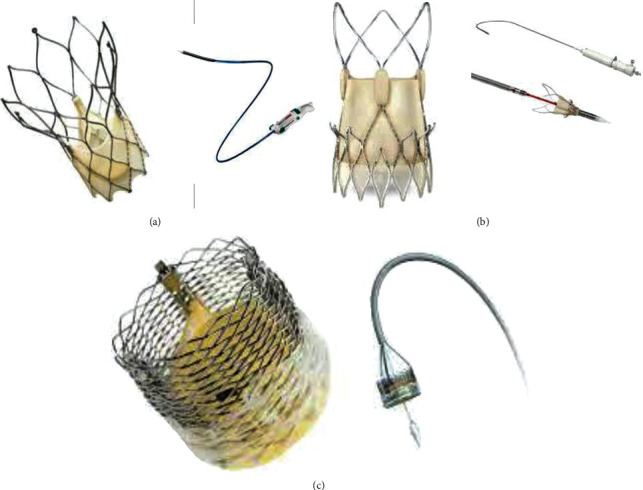
Last generation of THV. (a) ^ℷ^The Portico resheathable transcatheter aortic valve system (Abbott Structural Heart, St Paul, MN, USA). (b) ^†^The ACURATE neo (Boston Scientific, Marlborough, MA, USA) self-expanding THV. (c) ∗Lotus mechanically expanded valve (Lotus Valve System (MEV; Boston Scientific Corp). (a) Portico valve is designed with large, open cells, and intra-annular leaflet placement to preserve flow and access to the coronary arteries after deployment. The Portico valve was delivered by a flexible, first-generation Portico Delivery system, which had an 18F outer diameter for the small valves (23 and 25 mm) and a 19F outer diameter for the larger valves (27 and 29 mm). (b) The ACURATE neo bioprosthesis consists a self-expanding nitinol frame with three porcine pericardial leaflets and a stent body with an outer and inner pericardial skirt. (c) The MEV combines 3 bovine pericardial tissue valve leaflets and a braided nitinol frame with a polycarbonate-based urethane adaptive seal.

The Portico valve is composed of three intra-annular, bovine pericardial tissue valve leaflets mounted in a radiopaque, nonflared, self-expanding nitinol frame with a porcine pericardial valve sealing cuff [81] (Figure 7(a)). The ACURATE neo bioprosthesis combines a self-expanding nitinol frame with three porcine pericardial leaflets and a stent body with an outer and inner pericardial skirt [82] (Figure 7(b)).  Figure 7(c) repots a Lotus mechanically expanded valve (Lotus Valve System (MEV; Boston Scientific Corp).

A very low rate of infection was described in the pilot PARTNER randomized trial [74–77].

The landmark study from Reguiero et al. [83] is the first investigating systematically the issue of valve endocarditis after TAVR procedures in a large cohort including patients from several centers 1. Within the all-study cohort, 250 patients developed definite infective endocarditis after TAVI (1.24%), while in the individual data cohort of 6398 patients from 31 sites, 108 patients were diagnosed with endocarditis (1.68%). The causative pathogens that spread on the surface of biomaterial and frame stent were *enterococci strains* in 24.6% and S. aureus in 23.3%.

Sixteen centers (13608 patients) declined to participate with individual data contributing to a significant bias in the understanding of results. Another potential bias could arise by the recruitment of centres from very different health care systems across the world. The difference in clinical approaches, treatment protocols, postoperative management, and home-care assistance play an important role in the development of postoperative infections. The only parameter directly related to the procedure predicting endocarditis risk was aortic regurgitation, but the lack of intermediate data between TAVR and rehospitalisation prevents a full understanding of the pathological evolution. Worsening of NYHA class, rehospitalisation for heart failure, or dialysis would provide important information, especially for primary-care physicians to refer the patient and avoid triggers for infection. Endocarditis patients were younger, reflecting the current expansion to lower-risk candidates of TAVR. The in-hospital mortality rate was 36%, and surgery was performed only in 14.8% of the patients during the infective endocarditis episode. In survivors, overall mortality at 2 years was as high as 67%. These data outnumber the ones on surgical mortality for prosthetic valve endocarditis after surgical aortic valve replacement [84] and on mortality after endocarditis treatment. Again, little information is provided on the intervened conditions rendering these patients unsuitable for surgery and on the decision making for a conservative management of these patients although a high percentage of them had at least 1 indication for operation. Authors concluded that surgery was unable to influence the mortality rate of this cohort but operation has been actually offered to a potentially nonrepresentative portion of the cohort.

Interestingly, this study arrives timely after the one from Hansson et al. [85] reporting a 7% rate in risk of thrombosis after TAVR [3]. The inability of TAVR to deal with annular calcification seems to be at the root of residual regurgitation and paravalvular leaks, which are responsible of hemodynamic alterations that further result in thrombosis and subsequently endocarditis.

A multicenter study [6] reported an infection incidence of 0.5% in the first year after the percutaneous procedure. The pathogens responsible for the infection were for the majority of cases the most common strains of staphylococci (*Staphylococcus CoNS* 25%; *Staphylococcus aureus* 21%) or *enterococci* (21%). The colonization of the device by pathogens was indifferently localized on the stent frame, the leaflets, or both components. It should be noted that antibiotic prophylaxis was used in 59% of the infected and that although the self-expanding CoreValve system (Medtronic, Minneapolis, MN) was an independent risk factor for IE (hazard ratio [HR]: 3.1; CI 95%: 1.37 to 7.14), this deserves further evaluation for validation.

Another study [7] reported results on 55 patients with endocarditis after post-TAVI with an incidence of 3.02%. In 42% of cases, the infection of the materials was due to clinical treatment carried out after the patients were discharged. Multivariate analysis showed that device infections were related to the high comorbidity of the recipients of the device such as chronic hemodialysis and peripheral arterial disease. The pathogens detected at the infection sites were *Staphylococcus aureus* in 38% of cases, *enterococci* in 31%, *Staphylococcus CoNS* in 9%, and *streptococci* in 9.1% of cases.

None of the materials assembled in the devices described [74–82] seems to be exempt from the possibility of manifesting an infectious process. For the most recent randomized trials, the results reporting the development of infection are very scarce to draw definite conclusions [74–82].

### 5.2. Cardiac Implantable Electronic Devices

Cardiac device infections (CDI) have increased in proportion, distinguishing healthcare in the 21st century from the 20th [86–88]. The infection arises after the insertion of cardiac implantable electronic devices (CIEDs), which include permanent pacemakers, implantable cardioverter defibrillators, and cardiac resynchronization therapy devices. The spread of the pathogen can originate from the material implanted in the right cavities and evolve as tricuspid valve endocarditis (TVE) leading to infectious involvement of one or more TV leaflets [86, 87]. A large number of patients, despite respiratory symptoms caused by pulmonary embolism, pneumonia, and lung abscess formation, can successfully be managed clinically without resorting to surgery.

It is important to underline the disproportion that exists between the number of CDI reported in the United States (from 1 to 10 per 1,000 device/years) compared to the corresponding increase in the rate of implanted devices, thus, recording a large tendency for materials to develop infection [21, 22, 86–88]. The progression towards increased CDI leads to two main considerations. First, rapid removal of infected materials is recommended to avoid progression of infection to clinically more severe disease leading to a noticeable increase in short- and long-term mortality and morbidity. Second, infections incur an incremental cost of ownership calculated at over $ 15,000 per patient while taking into account the different rates of infection that vary according to the different types of implanted devices (approximately 1 per 1,000 device-years per pacemaker and 8 at 9 per 1,000 device years for complex devices) [22].

Endocarditis occurring from the CIED usually involves the generator pocket and subsequently progresses to the generator leads. From these, if not promptly counteracted, it can extend to the valvular and nonvalvular endocardial surfaces. Commonly, the initial inflammatory process is characterized by cellulitis or erythema and is limited to the pocket containing the generator. Its evolution as a widespread infection sees the involvement of the entire device whereby eradication is very difficult. The infected material and evident erosion of the skin overlying the pocket highlight a considerable risk of haematogenous spread of the infection.

*Staphylococci* belonging to the *CoNS strain* are the leading pathogens responsible for infection between 60% and 80%. *Enterococcus faecalis* is the most common causative pathogen in patients with digestive system cancer for whom CIED implantation is required [87–89]. In these patients, the vena cava is the most common route of entry [21, 22].

To avoid the onset of endocarditis of CIED, the use of antibiotic therapy as prophylaxis is recommended by evidence reported in both RCTs and observational studies. Prolonged use of antibiotic administration may be required in conjunction with negative serial blood cultures for 72 hours prior to reimplantation if the use of a new device is required [90, 91].

Treatment of CIED-related right-sided endocarditis is an emerging field [21, 92, 93], and recent studies agree that both delayed decision making and lack of experience in treating the disease can lead to intraprocedural and postoperative complications [94]. Surgery is aimed at removing the infection, restoring tricuspid valve competence, and reducing the risk of bacterial pulmonary embolism during the removal of the infection.

The severity of the infection guides the choice of procedure. Involvement limited to leads may involve simple extraction of the infected material which can be performed percutaneously [92, 95]. However, it is recommended that these procedures be performed in the presence of a cardiac surgeon as the risk of right ventricle rupture, whether multiple catheters are removed or if long-term devices are implanted, is high requiring immediate sternotomy and surgical repair. The presence of vegetations or damage to the tricuspid valve requires the use of a standard valve-related surgical procedure including vegetectomy, leaflet repair with a pericardial patch, tricuspid valve repair, or replacement.

Considering the worrying epidemiology of the problem and the causal etiology, two principles emerge for an adequate management strategy of CIED-related tricuspid valve endocarditis. First, the creation of a team dedicated to endocarditis made up of surgeons and cardiologists to avoid delays in diagnosis. Second, treatment with a stricter antibiotic prophylaxis policy is aimed at protecting high-risk patients and multidrug-resistant organisms [96].

Another growing subgroup of patients at risk for tricuspid valve endocarditis are individuals with end-stage renal disease for whom vascular access and CIED are required. For these patients, the risk reduction strategy involves performing arteriovenous fistulas on the contralateral upper limb to allow for the placement of an implantable cardiac electronic device so as to avoid the introduction of the central venous catheter. The increasing use of miniaturized CIEDs and even leadless pacemakers/implants is an important prospect for patients with rapidly progressing chronic kidney disease [97].

Importantly, the studies by Polewczyk et al. [98, 99] stressed that all types of CIED infections were associated primarily with procedure-related factors, while long-term mortality was caused by clinical factors. The heterogeneity of factors influencing the development of isolated pocket infection and isolated lead-related infective endocarditis or both confirms that 2 CIED infection variants were required.

In this direction, a benefit derives from treatment with anticoagulant and antiplatelet drugs. Therefore, the efficacy of chronic anticoagulant therapy and antiplatelet treatment should lead us to consider this medical treatment as indispensable choice for the prevention of CIED infection.

## 6. Conclusion

Infective endocarditis continues to be a disease with significant morbidity and mortality. With the increasing use of implantable valves or devices, there has been a shift in the treatment and prevention strategies of these infections. Treatment often requires input from the multidisciplinary team due to the complex nature of the presenting patients. Antibiotic prophylaxis may have a role in reducing the incidence of these infections but further trials are needed to substantiate findings from previous studies.

## Data Availability

This is a review type manuscript. No privacy-infringing data was used for the manuscript.

## Abbreviation

MEV: Mechanically expanded valve
THV: Transcatheter heart valve.
